# Carotenoids

**DOI:** 10.3390/antiox8110516

**Published:** 2019-10-29

**Authors:** Volker Böhm

**Affiliations:** Institute of Nutritional Sciences, Friedrich Schiller University, Dornburger Str. 25-29, 07743 Jena, Germany; volker.boehm@uni-jena.de

Carotenoids are a group of natural pigments, consisting of more than 750 compounds known so far. Their colours are mostly yellow, orange, or red due to the system of conjugated double bonds. This structural element is also responsible for the good antioxidant properties of many carotenoids. Carotenoids have shown numerous biological activities (not only as provitamin A), making them an interesting topic for researchers of various disciplines all over the world looking for, e.g., preventive properties of fruits and vegetables. As lipophilic compounds, their uptake and storage in the body are dependent on various conditions. In vitro and in vivo data showed stimulating and inhibitory effects of matrix compounds on bioaccessibility and bioavailability of carotenoids [1].

This special issue highlights some of the recent advances in carotenoid research, showing on the one hand the status quo and giving on the other hand new insight in functions and physiological relevance. Al-Yafeai et al. investigated rosehips of *Rosa rugosa* at different degrees of ripeness and showed that maturity stage significantly affected contents of bioactive ingredients as well as antioxidant capacity. Thus, harvesting date can be chosen depending on the contents of bioactive molecules. In addition, the authors fully characterized *(5’Z)*-rubixanthin (gazaniaxanthin) as the main (*Z*)-isomer of rubixanthin in hips of *R. rugosa* by using HPLC-MS/MS and NMR [2]. Chitong et al. investigated biological activities of astaxanthin extracted from shrimp waste. They discussed a possible use of this extract in dietary supplements for skin health applications [3]. Apocarotenoids are cleavage products of carotenoids. These molecules often showed biological activity. Thus, Zoccali et al. extracted four microalgae strains and characterized the apocarotenoids therein [4]. Sandmann reviews structure dependence of antioxidant protection from UV and light stress by carotenoids. Substitutions at the hydrocarbon molecule are important for good singlet oxygen quenching and radical scavenging [5].

The review by Elvira-Torales et al. gives an overview on beneficial health-related effects of carotenoids with special emphasis on liver health. The authors described, e.g., how carotenoids can interact in patients with non-alcoholic fatty liver disease (NAFLD) [6]. Results of an experiment using Wistar rats are presented by Róvera Costa et al. The authors evaluated the liver protective effects of supplementation (10 weeks) with lycopene on non-alcoholic fatty liver disease [7].

Chambers et al. synthesized special retinoyl-flavonolignan hybrids and investigated their antioxidant properties [8]. The paper of Karpiński and Adamczak reports results of antimicrobial activity of the carotenoid fucoxanthin against 20 bacterial species [9]. Balić et al. review the photoprotective properties of carotenoids in skin. Dietary carotenoids accumulate in the epidermis and act as a protective barrier to various environmental influences [10].

In vitro digestion experiments with human milk were done by Xavier et al. These authors investigated how the lactation stage (colostrum, mature milk) affected the carotenoid contents in micelles [11]. Wang et al. prepared inclusion complexes of lycopene in β-cyclodextrin and characterized these complexes by looking for stability and antioxidant activity. Lycopene was embedded into the cavity of β-cyclodextrin with a 1:1 stoichiometry [12].

Thus, this special issue presents a lot of various results, highlighting preventive effects of carotenoids in diverse conditions. In addition, some properties of single carotenoids are presented to deepen the knowledge on this fascinating group of compounds.
